# Fibroblast Growth Factor 21 (FGF21) Protects against High Fat Diet Induced Inflammation and Islet Hyperplasia in Pancreas

**DOI:** 10.1371/journal.pone.0148252

**Published:** 2016-02-12

**Authors:** Garima Singhal, ffolliott Martin Fisher, Melissa J. Chee, Tze Guan Tan, Abdelfattah El Ouaamari, Andrew C. Adams, Robert Najarian, Rohit N. Kulkarni, Christophe Benoist, Jeffrey S. Flier, Eleftheria Maratos-Flier

**Affiliations:** 1 Division of Endocrinology, Beth Israel Deaconess Medical Center, Boston, Massachusetts, 02215, United States of America; 2 Department of Microbiology and Immunobiology, Harvard Medical School, Boston, Massachusetts, 02215, United States of America; 3 Section of Islet Cell Biology and Regenerative Medicine, Joslin Diabetes Center, Boston, Massachusetts, 02215, United States of America; 4 Lilly Research Laboratories, Diabetes Research, Indianapolis, Indiana, 46225, United States of America; 5 Department of Pathology, Beth Israel Deaconess Medical Center, Harvard Medical School, Boston, Massachusetts, 02215, United States of America; University of Valencia, SPAIN

## Abstract

Fibroblast growth factor 21 (FGF21) is an important endocrine metabolic regulator expressed in multiple tissues including liver and adipose tissue. Although highest levels of expression are in pancreas, little is known about the function of FGF21 in this tissue. In order to understand the physiology of FGF21 in the pancreas, we analyzed its expression and regulation in both acinar and islet tissues. We found that acinar tissue express 20-fold higher levels than that observed in islets. We also observed that pancreatic FGF21 is nutritionally regulated; a marked reduction in FGF21 expression was noted with fasting while obesity is associated with 3–4 fold higher expression. Acinar and islet cells are targets of FGF21, which when systemically administered, leads to phosphorylation of the downstream target ERK 1/2 in about half of acinar cells and a small subset of islet cells. Chronic, systemic FGF21 infusion down-regulates its own expression in the pancreas. Mice lacking FGF21 develop significant islet hyperplasia and periductal lymphocytic inflammation when fed with a high fat obesogenic diet. Inflammatory infiltrates consist of TCRb+ Thy1+ T lymphocytes with increased levels of Foxp3+ regulatory T cells. Increased levels of inflammatory cells were coupled with elevated expression of cytokines such as TNFα, IFNγ and IL1β. We conclude that FGF21 acts to limit islet hyperplasia and may also prevent pancreatic inflammation.

## Introduction

Fibroblast growth factor 21 (FGF21), a member of the FGF superfamily, has emerged as an important metabolic regulator. In liver, FGF21 is induced by fasting and consumption of a ketogenic diet and plays a key role in fatty acid oxidation [1–4]. FGF21 has protective roles in mice consuming a lipotoxic diet, in part by enhancing fatty acid activation and disposal [5]. In white (WAT) and brown adipose tissue (BAT), FGF21 regulates the response to cold exposure by activating thermogenic programs in both tissues [6–8]. In muscle and liver, FGF21 expression is induced under conditions of cellular and mitochondrial stress [9]. When administered systemically FGF21 leads to improved glucose homeostasis and weight loss and, therefore, the potential of FGF21-based therapies in humans is being actively explored [10–11].

High levels of FGF21 mRNA and protein are expressed in the pancreas: however the physiology of FGF21 in this tissue remains obscure. Thus far, limited studies suggest that FGF21 has a role in modulating inflammation and damage induced by experimental pancreatitis [12–13]. FGF21 null mice develop more damage than wild type mice, while mice overexpressing FGF21 show an attenuated phenotype [12]. Further studies identified the transcription factor MIST1 as an upstream regulator for FGF21 and deletion of *Mist1* gene leads to a marked reduction in pancreatic FGF21 levels by epigenetic silencing which results in increased susceptibility to pancreatitis [13].

FGF21 may play a role in enhanced islet transplant survival in a model of streptozotocin-induced diabetes [14]. FGF21 also promotes β-cell survival and protects isolated rat islets and insulin- producing INS cells from glucolipotoxicity and cytokine-induced apoptosis [15]. However, no effect of FGF21 on insulin or glucagon secretion from the islets isolated from healthy animals has been described [16]. In contrast, FGF21 stimulates insulin secretion in *ex vivo* islets isolated from diabetic animals [15], although islets from the obese diabetic *db/db* mouse fail to respond to FGF21, possibly as a consequence of reduced β-klotho expression [17].

While all of these studies suggest that pancreatic FGF21 has a protective role in acinar and endocrine tissue, little is known about the expression and regulation of FGF21 in this complex heterogeneous organ which consists of multiple types of specialized endocrine and exocrine cells. Therefore, we aimed to better understand the regulatory physiology of pancreatic FGF21 in mice. We found that FGF21 is abundant in the pancreas and is largely derived from expression in acinar tissue. FGF21 expression in exocrine pancreas is regulated at both the protein and mRNA level. In contrast, mRNA expression in islets is very low and does not appear to be regulated. Systemic injection of FGF21 induces ERK1/2 phosphorylation (pERK 1/2) in 50% of acinar cells as well as in some non-insulin secreting islet cells.

Further evidence for FGF21 action in the pancreas comes from the severe islet hyperplasia observed in mice lacking FGF21 and fed a high fat diet for 16 weeks. These animals also develop large inflammatory infiltrates in the periductal regions which were largely lymphocytic in nature. These combined data demonstrate specificity of expression and regulation of FGF21 in the pancreas and indicate that it is involved in limiting both inflammation and islet hyperplasia.

## Material and Methods

### Animals

All experiments were carried out using male C57BL/6 mice obtained from The Jackson Laboratory (Bar Harbor, ME) and maintained on a 12:12-hr light–dark cycle and an ambient temperature of 22°C ± 2°C. FGF21 deficient (FGF21 KO) mice were generated at Lilly Research Laboratories, Indianapolis, Indiana by a targeted disruption of the FGF21 locus and is previously described (4). Founder mice were subsequently backcrossed onto the C57BL/6 line at least 10 times before the initiation of these studies. At the time of the experiments, chow fed, WT mice were approximately 10 weeks old. For high-fat feeding studies, 24 week old WT and FGF21 KO male mice were fed either standard laboratory chow (5008; Lab Diet, St Louis, MO) or a commonly used obesogenic diet (fat content by calories 45%, sucrose 17%; Research Diets, New Brunswick, NJ) for 16 weeks until high fat diet fed animals achieve at least 10 grams excess weight over chow fed animals. Mice were acclimated to handling for a week before metabolic testing and were allowed *ad libitum* access to food unless otherwise stated. Body weights were recorded weekly, and glucose and insulin tolerance tests were performed during the last week of the feeding period as described below. Blood was collected for measurement of glucose and serum analysis levels at the end of the study. Pancreata were harvested and processed for various studies accordingly. All procedures were performed in accordance with National Institute of Health Guidelines for the Care and Use of Animals and were approved by the Beth Israel Deaconess Medical Center Institutional Animal Care and Use Committee.

### Recombinant FGF21 protein

Recombinant FGF21 protein was generated and generously provided by Lilly Research Laboratories (Indianapolis, IN). Human FGF21 was expressed in *Escherichia coli* and was subsequently refolded *in vitro* as previously described [18].

### Reagents

Antibodies were purchased from commercial vendors: FGF21 (AF3057; R&D systems, Minneapolis, MN); Insulin (ab7842; Abcam, Cambridge, MA); β-actin (ab8226; Abcam); α -tubulin (ab7291; Abcam); pERK1/2 (4370; Cell Signaling Technology, Denver, MA); ERK1/2 (9107; Cell Signaling Technology); glucagon (2654; Sigma Aldrich, St. Louis, MO); somatostatin (sc7819; Santa Cruz, Dallas, TX); CD3 (MA1-90582; Thermo Scientific, Waltham, MA). Collagenase type IV for islet isolation was purchased from Worthington biochemical (Lakewood, NJ). Bovine serum albumin and all other reagents were purchased from Sigma Aldrich (St. Louis, MO).

### Glucose and insulin tolerance tests

An intra-peritoneal glucose tolerance test (GTT) was performed in overnight-fasted mice. Blood samples were drawn from the tail vein before injecting 2 g/kg glucose and after 10, 20, 30, 60, 90 and 120 minutes. For measuring insulin levels in response to glucose stimulation, serum was collected before and after 15 and 30 minutes of glucose administration. An intra-peritoneal insulin tolerance test (ITT) was performed in animals after 5–6 hours of food restriction. Blood glucose levels were measured before and after 15, 30 and 60 minutes of insulin (1 U/kg; ip) administration. Glucose was measured using an automated glucose meter and insulin concentrations were measured by ELISA (Ultra Sensitive Mouse Insulin ELISA kit, Chrystal Chem, Downers Grove, IL, USA).

### *In vivo* FGF21 signaling

FGF21 was administered to anesthetized mice via the *inferior vena cava* (IVC) as previously described [19]. Briefly, mice were anesthetized using ketamine/xylazine cocktail via intraperitoneal injection (ip). The peritoneal cavity was then exposed via a lateral incision and either FGF21 (500 ng/gm body weight) or saline was directly injected into the IVC into a total volume of 30μl. Mice remained under deep anesthesia throughout the duration of the study. After 10 min, pancreas and perigonadal adipose tissue were dissected and either processed for immunofluorescence or flash frozen and stored at −80°C. Protein was extracted using radioimmunoprecipitation assay (RIPA) buffer and assessed using Western blot. After transfer, blots were probed using antibodies against ERK1/2 and pERK1/2.

### Immunohistochemical staining

Slides were deparaffinized with xylene and hydrated through an ethanol gradient and rinsed with phosphate-buffered saline (PBS). Antigen was retrieved by citric acid buffer (pH 6.0). Sections were subsequently treated with 0.3% H_2_O_2_ and washed by tris-buffered saline containing 1% Tween 20 (TBST) for 15 min followed by a 60 min blocking with normal goat serum (Vector Laboratories, Burlingame, CA), slides were incubated with anti-pERK1/2 antibody (1:200) overnight at 4°C. Slides were washed and incubated with a secondary antibody (1:1000, 1hr) at RT, and washed again with PBS and were incubated in ABC diluted 1:500 (Vectastain Elite ABC kit; Vector Labs) in PBS for 30 min, and rinsed in PBS. The sections were reacted with diaminobenzidine (DAB Peroxidase substrate kit; Vector Labs) for 3 min, and the reaction was quenched by submerging the sections in double distilled water, counterstained with haematoxylin. Stained sections were mounted on SuperFrost Plus slides (Fisher Scientific, Waltham, MA), air-dried, dehydrated in increasing concentrations of ethanol (50%, 70%, 95%, 100%), cleared overnight in xylene, then coverslipped with Permaslip (Alban Scientific, St. Louis, MO). The slides were mounted and analyzed with a Zeiss Axioplan light microscope and the images were acquired using AxioImager software (Carl Zeiss, Thornwood, NY). Total number of acinar cells were counted in each section and were quantified as percentage of total pERK-immunopositive acinar cells by a blinded observer.

### Immunofluorescence staining

Pancreata were dissected and fixed in Z-fix (Anatech Ltd. MI, USA) overnight, followed by immersing them in 30% sucrose for another 24 hrs. Tissues were then frozen and fixed in Optimal Cutting Termperature (OCT) medium using an ethanol/dry ice bath, and subsequently cryosectioned (5μm) for double immunostaining. Frozen sections were fixed with ice cold methanol for 10 min followed by blocking (5% normal goat serum, 0.1% Triton X 100) for 1 hr. Sections were incubated overnight (4°C) with anti-pERK1/2 (1:100) and either anti-glucagon (1:200) or anti-somatostatin (1:200) antibodies. Sections were then incubated (1h, RT) with fluorescent secondary antibodies conjugated with AlexaFluor-488 (Molecular Probes, Eugene, Oregon) for pERK1/2-immunoreactivity and AlexaFluor-594 (Molecular Probes, Eugene, OR) for glucagon, somatostatin and insulin-immunoreactivity. Co-immunostaining for pERK1/2 and insulin cells was performed sequentially. Sections single-labelled for pERK1/2-immunofluorescence was incubated with anti-insulin antibody (1:1000, 1hr, RT) followed by the fluorescent secondary antibody (1h, RT).

All samples were counterstained with DAPI (Sigma Aldrich). Photomicrographs containing three fluorescent color channels were obtained using a Zeiss Imager confocal microscope to acquire three-color samples; the excitation light (and emission filter) was provided by the Argon488 (band-pass (BP) 505–530 nm), HeNe543 (long-pass (LP) 560 nm) and Diode405-30 laser (LP 420nm) to capture AlexaFluor-488, AlexaFluor-594 and DAPI fluorescence, respectively. Confocal images were acquired as confocal stacks using PASCAL software (Carl Zeiss) then flattened using the LSM 510 Browser (Carl Zeiss).

Cell counts were performed using photomicrographs obtained with a charge-coupled device camera, and the β-cells, α-cells and δ-cells were quantified using ImageJ software (National Institute of Health, MD, USA). Assessment of pERK1/2 co-immunoreactivity with pancreatic endocrine cells was obtained by counting the number of pERK1/2 immunopositive nuclei co-localizing with the respective cytoplasmic endocrine staining. At least 2,000 islet cell nuclei were counted per animal (4 mice/group), and data were expressed as percentage of glucagon-pERK1/2 + or somatostatin-pERK1/2 + endocrine cells.

### Quantitative RT-PCR

To best preserve mRNA from pancreas, whole pancreas was dissected and immediately homogenized in Qiazol (Qiagen, Chatsworth, CA). Total RNA was isolated using the RNeasy Lipid Tissue Kit (Qiagen); DNA digestion step was included to prevent contamination of genomic DNA. Quality of RNA was checked by measuring 260/280 and 230/280 ratios using nanodrop (ND-1000 spectrophotometer, Thermo Scientific), a ratio of ~2 were considered pure. Complementary DNA was synthesized from 0.5 μg of RNA using a mixture of oligo (dT) and random hexamer primers with Quantiscript Reverse Transcriptase (QuantiTect Reverse Transcription Kit; Qiagen). Quantitative polymerase chain reaction was performed using a 7800HT thermal cycler (Applied Biosystems, Foster City, CA) and SYBR green master mix (Applied Biosystems). Expression of each target gene was quantified by transformation against a standard curve and normalized to cyclophilin gene expression. A comparative standard curve was generated from a randomized mix of control sample RNA and cDNA generated at concentrations 1X, 0.3X, 0.1X, 0.03X and 0.01X compared to sample cDNA concentrations. Integrity and purity of cDNA was again confirmed by consistence of Ct values of housekeeping genes. Primers were designed using Primer3 online software (Open Source) and obtained from Invitrogen (Carlsbad, CA) [20]. Primer sequences are available on request.

### Immunoblotting

In brief, tissues were homogenized in RIPA buffer (150 mm NaCl, 1.0% Nonidet P-40, 0.5% sodium deoxycholate, 0.1% sodium dodecyl sulfate, 50 mm Tris, pH 8.0) supplemented with a Complete Mini Protease Inhibitor Cocktail (Roche, Indianapolis, Indiana) and phosphatase inhibitors. Protein concentrations were determined with a Bio-Rad protein assay (Bio-Rad Laboratories, Hercules, CA). Protein (20 μg) was analyzed by SDS-PAGE on a 4–15% Criterion Tris/HCl gel (Bio-Rad Laboratories) and transferred onto a 45 μM nitrocellulose membrane (Protran; Schleicher & Schuell, Keene, NH). Blots were then probed with each specified primary antibody and an HRP-conjugated secondary antibody (Jackson Immunoresearch Laboratories, West Grove, PA). The protein bands were then visualized with Super Signal West Pico chemiluminescent reagent (Pierce Chemical Co., Rockford, IL) and subsequently quantified using Image quant TL8.1 (GE healthcare lifesciences, Pittsburgh, PA). Representative blots are shown and all experiments were repeated at least twice.

### Isolation of pancreatic islets

Islets were isolated as described previously [21] using the intraductal collagenase technique. Briefly, the common bile duct was cannulated in the anterograde direction and the pancreas distended with collagenase (type IV) (Worthington). Pancreas was harvested and digested for 25–30 min at 37°C. Islets were isolated by generating sedimentation gradient of histopaque 1077 (Sigma Aldrich). Floating islets were washed and healthy islets were hand-picked, and then transferred into RPMI 1640 (Fisher Scientific) islet culture medium containing 10% fetal bovine serum, 20 units/ml penicillin G, 20 g/ml streptomycin, 7 mmol/l glucose at 37°C in 5% CO_2_. Acinar fraction was collected by washing the pellet twice with PBS.

### *In Vitro* insulin and glucagon secretion

For *ex vivo* experiments, islets were washed with RPMI-1640 (3.3 mmol/l glucose; 10% fetal bovine serum, and penicillin-streptomycin complex) and cultured for 24 hr at 37°C before experiments. For secretion experiments, 15–25 mouse islets of similar sizes (120–150 μm) were handpicked from a single harvest pool and cultured for 24 hr. To evaluate the effects of FGF21 on insulin secretion, islets were stimulated with different concentrations of FGF21 (0, 10, 50, 100nM) for 1 hr at 3.3 and 20mmol/l glucose concentrations. Media were collected and centrifuged, and supernatants were stored at −20°C for insulin enzyme-linked immunosorbent assay (ELISA) or glucagon radioimmunoassay (RIA).

### Quantification of islet and inflammation surface area

Islet surface area was quantified by total β-cell area as described previously [21–22]. Briefly, pancreatic sections were stained for insulin to visualize β-cells and images were captured with an Olympus VS120 Slide scanner microscope. Each section was analyzed separately and the surface area was calculated using ImageJ software. Total islet surface area was expressed as a percentage of the total pancreatic surface area in the surrounding section. At least 3 different sections/animals were analyzed which were >100 μm apart. Percentage of inflammation was also quantified in a similar way by staining the sections with a CD3 antibody.

### Subcutaneous FGF21 infusion

FGF21 was delivered for 3 days using Alzet mini-osmotic pumps (Durect Corporation, Cupertino, CA). Pumps were filled with either FGF21 (1 μg/hr; 24 μg/day) or vehicle (saline) and primed for 4–6 hr at 37°C before implantation. The pump was inserted into the interscapular region under isoflurane anesthesia. Briefly, the surgical area was shaved and cleaned with povidone–iodine solution and an alcohol swab. A small lateral incision was made (10mm) and the pump was carefully inserted. The incision was closed with sutures, and mice were allowed to recover from anesthesia in their home cages.

### Isolation of lymphocytes from pancreas

Pancreata were harvested immediately after perfusion through the left cardiac ventricle and dissected free of contaminating lymph nodes or fat strands and placed into cold DMEM (Fisher Scientific). Pancreas were cut in small pieces and digested with freshly prepared digestion buffer; collagenase IV (1 mg/ml; C5138; Sigma Aldrich), DNase I (10 U/ml; DN25; Sigma Aldrich) and 1% FCS (30 min at 37°C) in a shaking water bath. The digested product was filtered through a 70 mm mesh using a plunger to disrupt undigested tissue and washed with DMEM supplemented with serum. To separate the leukocyte fraction, ACK lysis (Ammonium-chloride-potassium) (A10492-01; Thermo Scientific) was performed as manufacture’s instruction at room temperature and followed by centrifugation at 1200 rpm for 10 min. Pellet was washed again with cold DMEM containing FCS, and resuspended, and stained for analysis by flow cytometry.

### Flow cytometry

For immune population analysis, cells were stained in FACS buffer (PBS + 2% FCS + 1 mM EDTA) at 4°C for 20–30 min. Staining was performed using fluorochrome-conjugated antibodies for anti-CD45, -TCRb, -CD90.2, -CD19, -CD4, -CD8, -TCRgd, -NKp46, -CD11b, -CD11c, -F4/80, -I-A/E, -Ly6C (Biolegend, San Diego, CA); anti-Foxp3 (eBioscience, San Diego, CA). Fluorochromes were APC-Cy7, PE-Cy7, PE-texas red, pacific blue, Alexa fluor 700, FITC, PE and APC. Cells were washed twice with FACS buffer followed by centrifugation at 1500 rpm for 5 min and resuspension in the same buffer. To detect intracellular expression of Foxp3, cells were fixed and permeabilized using the Intracellular Fixation & Permeabilization buffer set (88–8824; eBioscience, San Diego, CA) according to the manufacturer’s protocol. Briefly, cells were resuspended in eBioscience Fix/Perm buffer for at least 30 min before washing and resuspended in Perm/Wash buffer containing antibody to Foxp3. Data was analyzed by Flowjo single cell analysis software (Flowjo LLC, Ashland, OR).

### Serum analysis

Blood samples were collected on ice, heparinized and spun at room temperature before storage of plasma at 4°C or allowed to clot and spun at 4°C before flash-freezing of serum in liquid nitrogen. Serum metabolites were measured by small-scale enzymatic assay for glucose (1070; Stanbio Laboratory, Boerne, TX) or using a standard automated glucose monitor. Serum insulin levels were determined by an ultrasensitive mouse-ELISA (Crystal Chem, Downers Grove, IL). Plasma levels for insulin like growth factor -1 (IGF-1) was determined using Quantikine ELISA kit for mouse/rat IGF-1(MG100; R&D Systems, MN, USA). Radioimmunoassay kit for glucagon estimation (GL-32K; Millipore, Billerica, MA) was used. For glucagon, blood samples were collected in tubes containing aprotinin (1 g/ml, Bayer Healthcare, Berkeley, CA), and the serum was stored at -80°C before radio-immunoassay. Serum amylase levels were analyzed using Biovision assay kit (K711-100; Biovision, Milpitas, CA).

### Statistical analysis

GraphPad Prism (La Jolla, CA) was utilized to analyze statistical differences using analysis of variance (ANOVA) test where appropriate. For comparisons of two groups, the Student’s t-test was used. Where it is shown in figures, *p < 0.05; **p < 0.01 and ***p < 0.001 indicate the levels of significance. Data are displayed as the mean ± Standard error of the mean (SEM).

## Results

### Pancreatic FGF21 is nutritionally regulated and is derived mainly from the acinar pancreas

**Fig 1:**
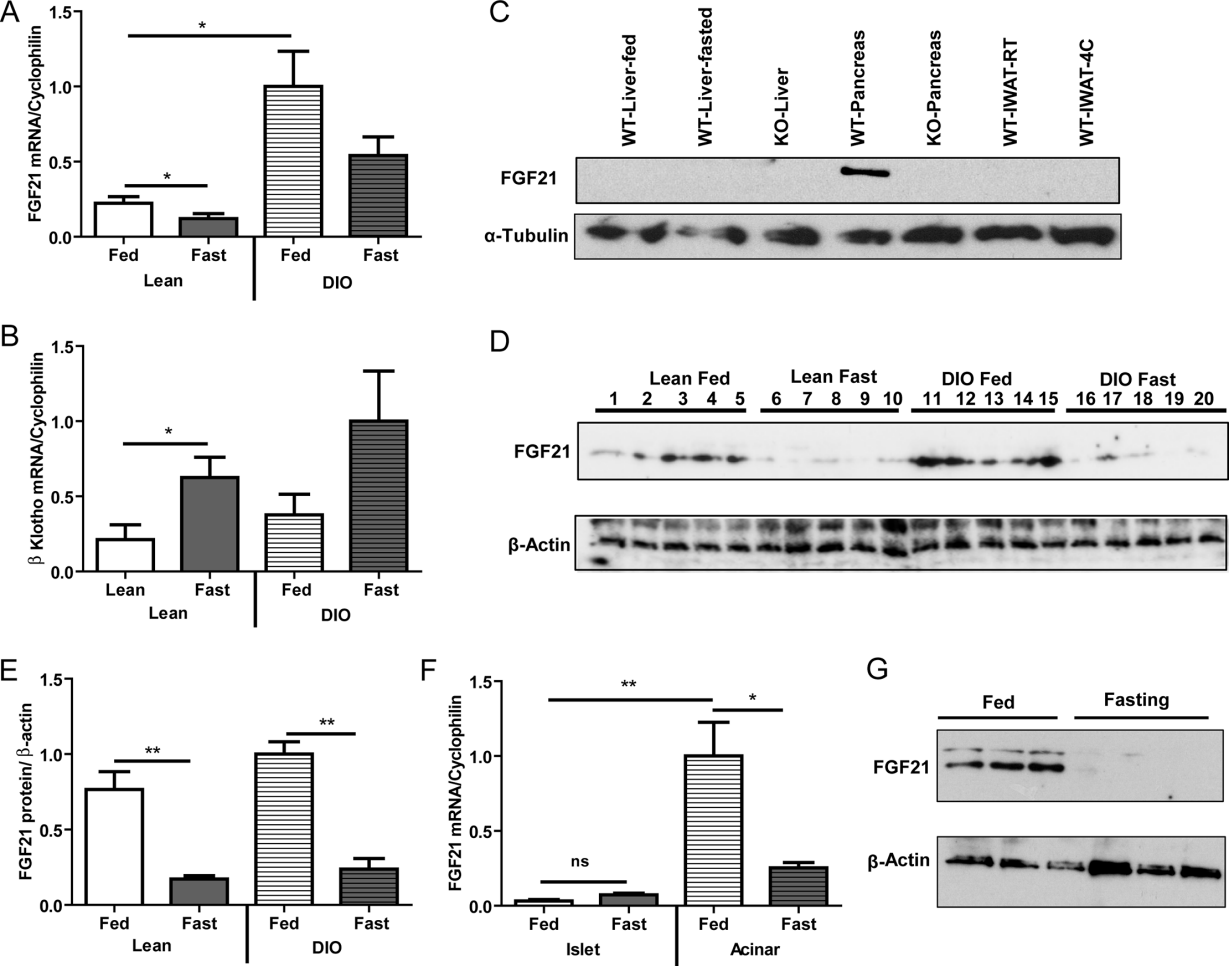
Expression and nutritional regulation of FGF21 in pancreas. FGF21 is regulated by the nutritional status in fed, fasted and diet-induced obesity (DIO). Fasting reduced FGF21 mRNA in whole pancreas (A). β-klotho, the co-receptor of FGF21, is expressed in the pancreas and is inversely regulated by fasting (B). FGF21 protein was only detected in pancreas compared to liver and inguinal white adipose tissue (IWAT) (C). Pancreas FGF21 protein level was reduced by fasting in both chow fed and high fat diet fed conditions (D, E). Separation of pancreatic fractions to evaluate the individual contribution of islets and acinar tissue on FGF21 expression show that acinar pancreas contributes to the majority of FGF21 levels while islet cells have very low FGF21 levels and are not nutritionally regulated (F). Acinar expression of FGF21 is regulated with fasting (F, G). n = 6 per group. Each western blot lane represents an individual animal.

In liver, FGF21 expression is nutritionally regulated [1–4], rising markedly with fasting. We therefore examined the possibility that nutritional status may also affect the expression of FGF21 in the pancreas. After a 24 hr fast in lean animals, a significant reduction of ~50% was seen in FGF21 mRNA levels (Fig 1A; Lean Fed 0.22 ± 0.04; Lean fasted 0.12 ± 0.03; p = 0.04). In mice with diet induced obesity (DIO), FGF21 mRNA expression was 4-fold higher than that seen in lean animals (Fig 1A; Lean Fed 0.22 ± 0.04; DIO fed 1.0 ± 0.23; p = 0.02) and a 50% fall was observed with fasting.

β-klotho, the obligate co-receptor for FGF21 is also present in the pancreas. Interestingly, β-klotho expression is regulated in an opposite manner to FGF21 (Fig 1B). β-klotho expression significantly increased in lean animals with fasting (Lean fed 0.21 ± 0.09; Lean fasted 0.62 ± 0.13; p = 0.01); similar trends were observed in fasted obese animals, however the change did not reach statistical significance (Fig 1B).

We compared protein content, of FGF21, in several different tissues including liver and WAT in which mRNA expression is specifically regulated. At the protein level, FGF21 was only detected in pancreas (Fig 1C). In this tissue, consistent with changes in mRNA expression, a dramatic reduction of approximately 75% in FGF21 protein levels was noted with fasting (Fig 1D and 1E; Lean fed 0.76 ± 0.12; Lean fasted 0.17 ± 0.02; p = 0.01). Although at the protein level, FGF21 was slightly increased in animals with diet induced obesity, a similar reduction in protein was observed with fasting (Fig 1D and 1E; DIO fed 1 ± 0.23; DIO fasted 0.08 ± 0.07; p = 0.003). Considering the heterogeneous nature of the pancreas, we evaluated differential FGF21 expression by isolating islets and acinar fractions of the pancreas (Fig 1F). The purity of the isolated fractions was verified by checking the expression of insulin, glucagon and amylase; insulin and glucagon expression was limited to the islet fraction while amylase was limited to the acinar fraction (S1A Fig).

FGF21 expression in the acinar fraction was 99-fold higher than in the islet fraction (Fig 1F; islets fed 0.03 ± 0.009; acinar fed 1.0 ± 0.22; p = 0.01). Consistent with our results in whole pancreas, FGF21 mRNA in acinar tissue from fasted animals was reduced by about 75% (Fig 1F; acinar fed 1.0 ± 0.22; acinar fast 0.25 ± 0.03; p = 0.01). In contrast, a small non-significant increase in FGF21 mRNA was seen in the islets. Acinar protein levels are exquisitely sensitive to fasting, as FGF21 falls to almost undetectable levels (Fig 1G). The expression of FGF21 in islets was not detectable at the protein level.

All fibroblast growth factor receptors, including FGFR1, FGFR2, FGFR3 and FGFR4 were detected in both acinar and islet fractions. FGFR1 is expressed predominantly in islets while FGFR3 is predominantly expressed in acinar tissue (S1B Fig).

### Pancreas is a direct target for FGF21 signaling

**Fig 2:**
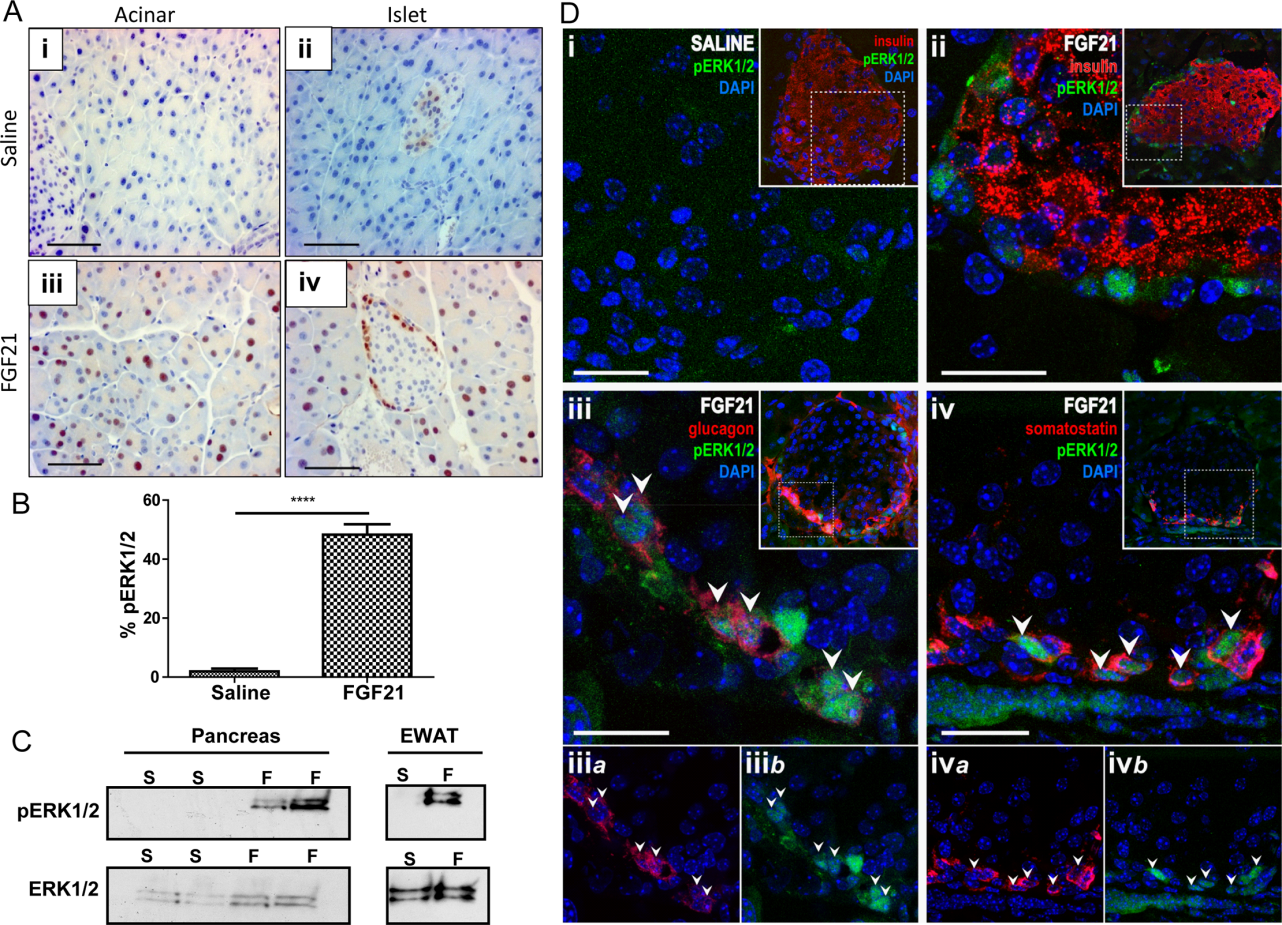
Induction of ERK1/2 phosphorylation in pancreas with acute FGF21 exposure and Co-immunostaining with endocrine hormones. Acute FGF21 exposure induces ERK1/2 signaling events therefore phosphorylation of ERK1/2 (pERK1/2) has been chosen as a marker of FGF21 signaling. Up to 50% of acinar cells show strong nuclear pERK1/2 staining (brown) after FGF21 administration (A i, iii). Islets represent a very unique pattern with pERK1/2 only on the peripheral cells (A ii, iv). Percentage of pERK1/2-labeled acinar cells with saline and FGF21 administration (B). Western blot of pERK1/2 and total ERK1/2 from the same pancreas samples (C). Epididymal white adipose tissue (EWAT) is a positive control for FGF21 signaling (C). S = saline and F = FGF21. Confocal photomicrographs show FGF21-induced pERK1/2 immunoreactivity in islets (D). Absence of pERK1/2 staining (green) after saline treatment (Di; *Inset*, islet outline is indicated by insulin-immunoreactivity shown in red). FGF21 did not elicit pERK1/2 in insulin (red)-producing β-cells (D ii). Merged images show pERK1/2 co-localization in glucagon-positive α cells (D iii) and somatostatin-positive δ cell (D iv). Split panels show glucagon (iii*a*) and pERK (iii*b*) labeling or somatostatin (iv*a*) and pERK (iv*b*) labeling in single cells. A robust and specific pERK1/2 staining was observed in almost 10% of total islet nuclei mostly at the periphery. Outlined region (dashed line) in the inset indicates the region represented in each panel. Representative pERK1/2 labelled cells are indicated by white arrows. n = 4 per group. Scale: 200 μm, A; 20 μm, D.

To demonstrate the acute and direct signaling by FGF21 on the pancreas, *in vivo*, we tested the ability of FGF21 to activate FGF signaling pathways. FGF21 was injected -directly into the *inferior vena cava*, and pancreatic tissue was harvested after 10 min and immunostained for pERK1/2 as a reporter of FGF21 signaling (Fig 2A). Compared to saline treatment (Fig 2A i-ii), acute exogenous FGF21 induced ERK1/2 phosphorylation (pERK1/2) in 50% of acinar cells (Fig 2A iii and 2B). In the islet, pERK1/2 was limited to cells located along the islet periphery (Fig 2A iv). Furthermore, we noted very strong nuclear staining consistent with pERK1/2 migration to the nucleus upon phosphorylation and activation. Using epididymal white adipose tissue (EWAT), a known target of FGF21 action, as a positive control, demonstrated enriched induction of pERK1/2 with FGF21 treatment (Fig 2C).

In mice, β-cells form the inner core of an islet which is surrounded by the other 4 types of endocrine cells at the periphery. These peripheral cells consist of glucagon-secreting α cells (15–20%), somatostatin-secreting δ cells (3–10%), pancreatic polypeptide (PP)-secreting PP cells (3–5%) and ghrelin secreting-ε cells (<1%) [23]. In order to identify FGF21-sensitive cells, we determined the co-localization between pERK1/2 and insulin, glucagon and somatostatin, respectively after 10 min of FGF21 treatment. FGF21 treatment resulted in a few, rare pERK1/2 positive β-cells in the inner core of the islet (Fig 2D ii). However at the islet periphery, glucagon-positive α-cells (Fig 2D iii*a*) and somatostatin-positive δ-cells (Fig 2D iv*a*) co-localized with pERK1/2 positive staining (Fig 2D iii-iv*b*). Quantification revealed that approximately ~10% of total islets cells were positive for pERK1/2 (Total islet nuclei 137 ± 18; pERK1/2+, 13 ± 2.3) while ~50% of glucagon cells (pERK1/2+ 11.1 ± 1.4; glucagon+pERK1/2+, 5.0 ± 0.9) and 30–40% somatostatin cells (pERK1/2+ 10.6 ± 1.1; somatostatin+pERK1/2+, 3.1 ± 0.7) were positive for pERK1/2. We did not observe any significant alteration in signaling pattern at later time points such as 20 minutes (data not shown).

Possible functional effects of FGF21 were examined using freshly isolated cultured primary islets. FGF21-stimulated insulin and glucagon release was measured in the presence of high or low glucose concentrations. At 3 mmol/l glucose, 50 nM FGF21 was associated with a small increase in insulin. No change was seen in the presence of high glucose (20 mmol/l) (S2A Fig). Additionally, FGF21 had no effect on glucagon release (S2B Fig).

### Exogenous treatment of FGF21 down-regulates its own expression in pancreas

**Fig 3:**
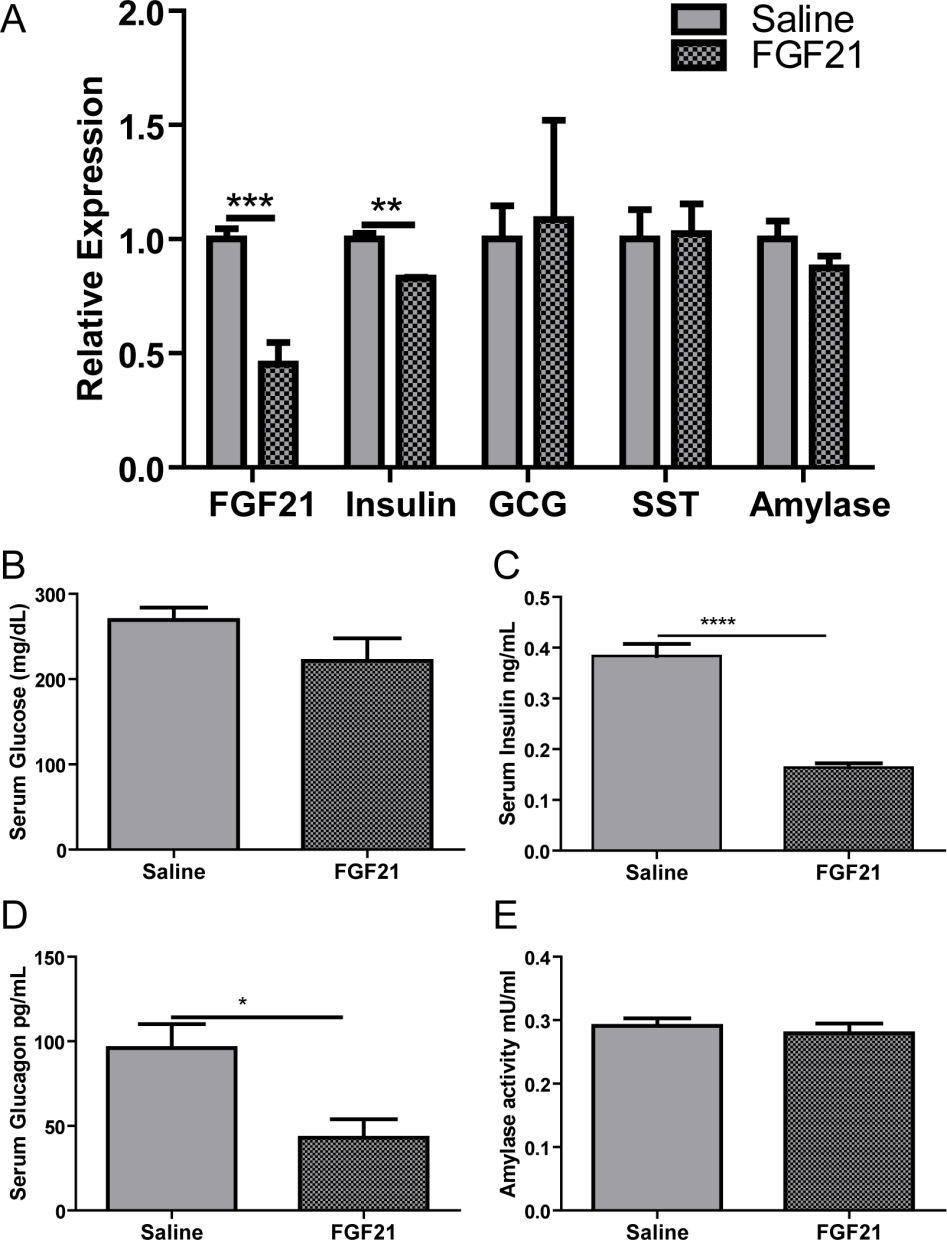
Effect of exogenous treatment of FGF21 in pancreas. Chronic FGF21 treatment for 3 days by mini osmotic pumps led to the reduction in FGF21 expression in pancreas (A). FGF21 infusion down-regulated insulin but glucagon, somatostatin or amylase expression. While FGF21 infusion did not alter serum glucose levels (B), it reduced the serum levels of pancreatic hormones, insulin (C) and glucagon (D) without affecting the amylase activity (E). n = 10 per group.

We evaluated changes in total pancreatic gene expression following 3 days of FGF21 infusion in mice using mini osmotic Alzet pumps. FGF21 mRNA levels was decreased by 50% (Fig 3A; saline 1.0 ± 0.04; FGF21 0.45 ± 0.09, p = 0.0008) suggesting exogenous FGF21 acts as a negative regulator of its own expression in the pancreas. FGF21 infusion did not change serum glucose levels significantly (Fig 3B) but down-regulated insulin mRNA expression (Fig 3A; saline 1.0 ± 0.02; FGF21 0.82 ± 0.003, p = 0.003) and reduced serum insulin (Fig 3C; saline 0.38 ± 0.02; FGF21 0.16 ± 0.008, p<0.0001). Expression of glucagon and somatostatin were unchanged (Fig 3A), however serum glucagon levels were reduced by FGF21 infusions (Fig 3D; saline 96.1 ± 14.2; FGF21 43.1 ± 10.8, p = 0.01). Pancreatic expression of amylase and serum amylase activity did not change in response to FGF21 infusion (Fig 3A and 3E).

### Long term high fat diet feeding caused severe islet hyperplasia in FGF21 KO animals

**Fig 4:**
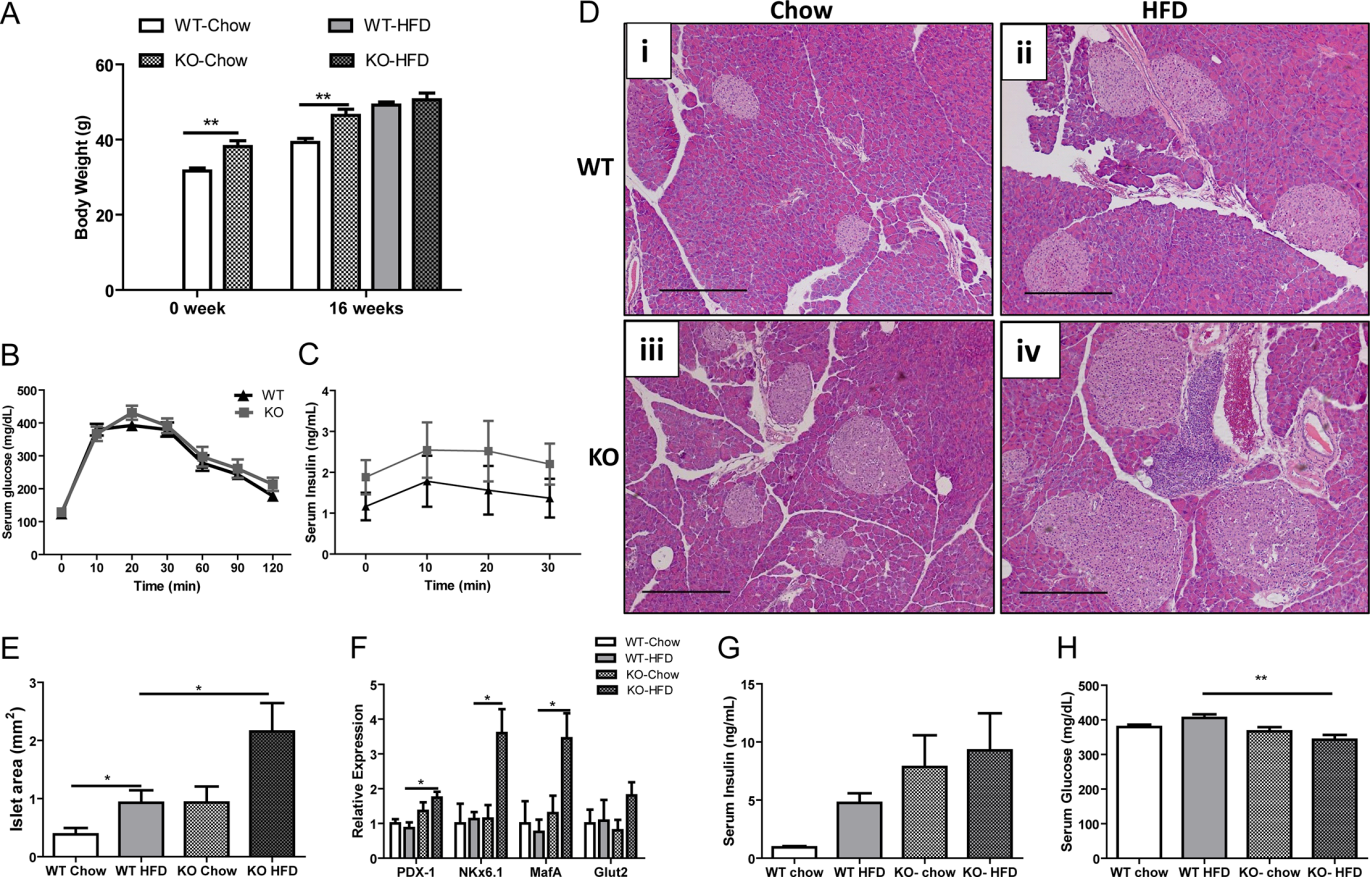
Long term high fat diet feeding caused severe islet hyperplasia in FGF21 KO animals. WT and FGF21 KO mice on chow diet have similar body weight (A), glucose tolerance (B) and insulin secretion in response to glucose (C), despite notable differences in their pancreatic morphology (D i, iii). After consuming high fat diet for 16 weeks, FGF21 KO mice (D iv) had increased islet hyperplasia compared with WT mice (D ii). Islet cell surface area was measured and represented (E). Islet cell proliferation markers were substantially over expressed in obese FGF21 KO animals (F). Islet hyperplasia leads to increased serum insulin (G) in FGF21 KO animals with a slight reduction in blood glucose (H) (n = 7 per group). Scale: 1 mm.

At a young age (8–10 weeks), WT and FGF21 KO animals do not exhibit any significant phenotypic or morphological differences in their pancreas, as determined by β-cell mass and islet cell surface area (S3A & S3B Fig). However, at 24 weeks (Fig 4A; WT Chow 31.7 ± 0.7; KO-Chow 38.2 ± 1.5, p = 0.002) and at 40 weeks (Fig 4A; WT Chow 39.3 ± 1.1; KO-Chow 46.4 ± 1.6, p = 0.003) chow fed FGF21 KO mice weighed more than WT mice. Interestingly, the differences in body weights between groups converged after high fat diet feeding for 16 weeks (Fig 4A). Glucose sensitivity in WT and FGF21 KO animals on high fat diet feeding was comparable as measured by glucose tolerance test (Fig 4B). Insulin secretion was also measured in these animals under glucose stimulation. Baseline insulin levels were slightly elevated in FGF21 KO mice, however, no significant differences were observed between WT and KO animals (Fig 4C).

To evaluate pancreatic morphology, we analyzed the sections of pancreas from WT and FGF21 KO animals consuming chow or high fat diet. Compared to chow fed animals (Fig 4D i) high fat diet fed WT animals developed islet hyperplasia with a 2-fold increase in islet size (Fig 4D ii and 4E). Interestingly, chow fed FGF21 KO animals showed a similar degree of islet hyperplasia to high fat fed WT mice at this age (Fig 4D ii-iii and 4E). Furthermore, obese FGF21 KO animals on a high fat diet developed severe islet hyperplasia (Fig 4D iv and 4E). Quantification revealed that FGF21 KO animals exhibited a 2-fold increase in islet area compared to WT high fat diet group (Fig 4E; WT-HFD 0.92 ± 0.21; KO-HFD 2.2 ± 0.48, p = 0.02). Increased islet cell surface area was associated with an increased expression of β-cell proliferation markers, including pancreatic and duodenal homeobox 1 (PDX-1), NK homeobox factor 6.1 (NKx6.1) and musculoaponeurotic fibrosarcoma oncogene family protein A (MafA) (Fig 4F). Circulating serum insulin levels were elevated in the obese FGF21 KO animals (Fig 4G) with a mild reduction in the serum glucose levels (Fig 4H).

### FGF21 KO animals demonstrate large perivascular lymphocytic inflammation on obesogenic diet

**Fig 5:**
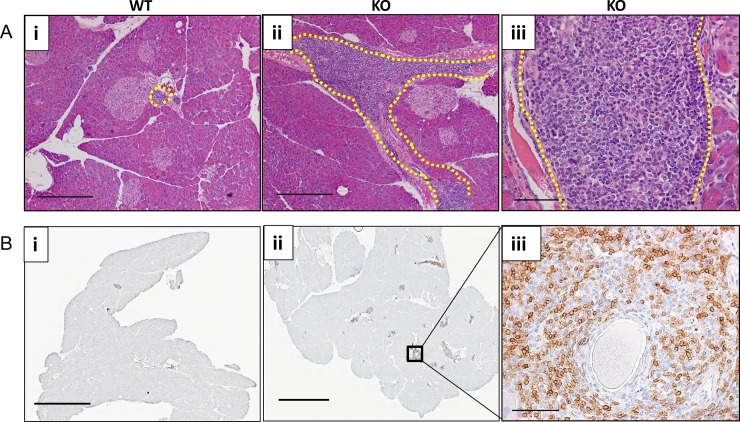
FGF21 KO animals demonstrate large perivascular inflammation on obesogenic diet. HFD consumption caused severe pancreatic inflammation in FGF21 KO mice (A). H&E staining showing, that compared to WT animals (i), FGF21 KO mice developed severe pancreatic periductal inflammation (outlined area in ii-iii) when consuming a HFD diet for 16 weeks. Lymphocytic nature of inflammatory cells is shown in a higher magnification image (iii). (B) Immunohistochemical analysis for the lymphocytic marker CD3 on the pancreas of WT (i) and FGF21 KO animals (ii-iii) is represented. FGF21 KO showed higher number of CD3, T cell receptor antigen. n = 7 per group. Scale: 1 mm, Ai-ii; 2 mm, Bi-ii; 200 μm, iii.

Less than 50% of WT animals on high fat diet had small focal areas of perivascular inflammation (Fig 5A i). However, large areas of inflammatory infiltrates were noted in all FGF21 KO animals on high fat diet (Fig 5A ii-iii). A high magnification image indicated that inflammatory cells exhibited lymphocytic features such as heterochromatic nuclei surrounded with a very thin layer of cytoplasm (Fig 5A iii). FGF21 KO animals eating chow had very mild inflammation (not shown). No inflammatory cells were observed in chow fed WT animals. In the FGF21 KO mice almost all the inflammation was present in close proximity to the pancreatic ducts (Fig 5A) and virtually no infiltration was observed in the islets.

The inflammatory pattern in FGF21 KO animals was not accompanied with significant fibrotic changes in the pancreas as observed by Masson’s trichrome staining (S4A Fig) and fibrotic gene expression markers remained unchanged (S4B Fig).

Immunostaining was performed to characterize lymphocytic markers in obese WT and FGF21 KO animals (Fig 5B). A large proportion of immune cells were positive for CD3 antigen, the cell surface marker for T lymphocytes (Fig 5B i-iii). Quantification of the surface area covered by CD3-labeled cells show that more than 10% of pancreas from FGF21 KO animals (CD3+ per total area 6.9 mm^2^/ 47.9 mm^2^) was covered by immune cells compared to less than 0.5% immune infiltration in WT pancreas (0.17mm^2^/46.6mm^2^).

**Fig 6:**
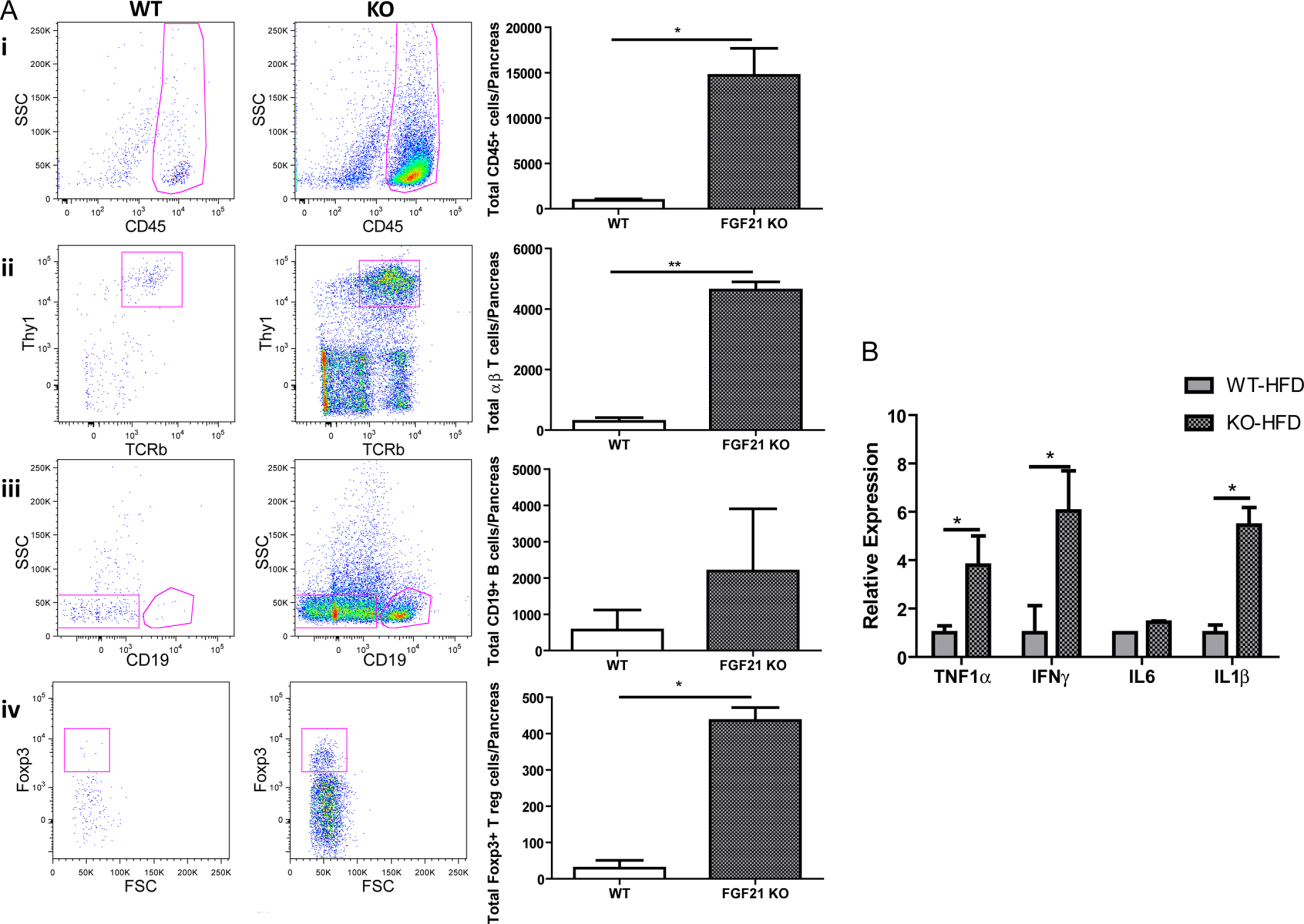
Predominant T lymphocytic inflammation in FGF21 KO obese mice. (A) Representative cytofluorometric dot plots of isolated lymphocytes from WT and FGF21 KO animals consuming high fat diet for 16 weeks. FGF21 KO mice show elevated CD45+ (i), TCRb+ and Thy1+ T lymphocytes (ii) and Foxp3+ Treg cells. CD19+ B lymphocytes were not significantly altered (iii). Corresponding summary data is shown in the right panel. The experiment was repeated twice (n = 4 per group). (B) Gene expression analysis of cytokines is presented. n = 7 per group.

To further characterize the infiltrating immune cells, pancreatic tissue of high fat diet fed WT and FGF21 KO animals were harvested and analyzed by flow cytometry (Fig 6). The common leukocyte marker, CD45, was used to quantitate the total number of immune cells. In FGF21 KO animals, a robust 15-fold increase in the total immune cell population was observed (Fig 6A i; WT HFD 933 ± 166, KO HFD 14685 ± 2983, p = 0.04). Further subtyping of the immune population confirmed a significantly higher proportion of TCRb+ Thy1+ T lymphocytes in FGF21 KO mice (Fig 6A ii; WT HFD 286 ± 125, KO HFD 4629 ± 270, p = 0.004) compared to CD19+ B lymphocytes (Fig 6A iii). We also characterized the immune infiltrate for other groups of lymphocytes such as γδ T cells, natural killer cells and NKp46 innate lymphoid cells. All these cell types were present at a slightly higher proportion in the FGF21 KO group but did not reach statistical significance (S5 Fig). Interestingly, the number of Foxp3+ T regulatory cells was 15-times elevated in FGF21 KO animals (Fig 6A iv, WT HFD 29.5 ± 21, KO HFD 436 ± 36, p = 0.01). A non-significant up regulation was also observed in the myeloid cells using common markers for dendritic cells, macrophages, and monocytes (S6A Fig). The expression of the most common membrane markers for myeloid cell lineages, such as CD68, F4/80 and CD36 were not altered in obese FGF21 KO animals (S6B Fig). However, a higher number of T lymphocytes was observed to be associated with higher levels of cytokines secreted by T cells such as TNF1α, IFNγ and IL1β (Fig 6B).

## Discussion

Our data demonstrate that in healthy *ad lib* fed animals, FGF21 expression is highest in the pancreas compared to all other organs. Indeed, at the protein level, given the sensitivity of currently available antibodies, western blot analysis only detects expression in the pancreas. Furthermore, we found that most pancreatic FGF21 derives from acinar tissue and that in comparison, expression in islets is negligible. Total pancreatic acinar expression changes with nutritional status, decreasing markedly in the fasted state which interestingly is opposite to that observed in liver where fasting leads to an increase in FGF21 expression. In contrast, FGF21 expression in islets remains unchanged during fasting. These findings reflect specific regulation of FGF21 in the pancreas, distinct from that seen in other tissues.

We also observed reduced expression of FGF21 following 3 days of peripheral FGF21 administration. During fasting, serum FGF21 is high as a consequence of increased hepatic expression of FGF21 and it is thus possible that the reduction of pancreatic FGF21, both in the fasted state and with exogenous FGF21 infusion, is a direct effect of FGF21 to down-regulate its own expression in the acinar cells. Peripheral administration of FGF21 in mice has also been seen to suppress its own expression in other tissues, i.e. WAT (unpublished data from EMF lab). Nevertheless, primary cell autonomous down-regulation in response to the nutritional status is also possible and can only be excluded with tissue specific ablation of FGF21.

Systemic administration of FGF21 stimulates ERK1/2 phosphorylation (pERK1/2) in pancreas, which is consistent with the expression of FGF receptors and β-klotho in this tissue. FGF21 induced pERK1/2 in half of acinar cells as well as in a subset of cells localized to the periphery of each islet. Within the islet, cells located at the periphery are largely glucagon-synthesizing α-cells and somatostatin-synthesizing δ-cells, in which we detected co-localization of pERK1/2 with somatostatin and glucagon. In contrast, co-localization was only rarely observed with insulin, thus suggesting that the β- cell is not an FGF21 target. While it appears that FGF21 targets at least a subset of endocrine cells within the islet, we detected no changes in secretion of either insulin or glucagon on healthy islets treated *ex-vivo* with FGF21 as also previously reported [15–16]. As long term FGF21 administration in mice down-regulates serum insulin and glucagon levels it seems likely that these systemic effects of FGF21 reflect the overall improved metabolic milieu of the animal i.e. from FGF21-mediated insulin-independent glucose uptake in adipose tissue [4, 24–25].

Despite the high levels of FGF21 in the acinar pancreas, the fact that FGF21 expression is regulated by both nutritional status and exogenous FGF21, and evidence showing multiple cell types respond to FGF21, young FGF21 KO animals had normal pancreatic histology. However, as the animals aged, islet surface area increased. Notably, when challenged with a high fat diet, older FGF21 KO mice developed significantly exacerbated islet hyperplasia compared to WT animals. Islet hyperplasia normally reflects a demand for more insulin and this is indeed what we observed, significantly elevated insulin levels in FGF21 KO mice consuming either diet. As FGF21 is known to stimulate glucose uptake it is likely that, over time, animals lacking FGF21 require greater levels of insulin to compensate. Although the absence of FGF21 in acinar tissue may specifically contribute to this phenomenon, it is unlikely because detailed studies have demonstrated the presence of distinct acinar and islets circulatory systems, with no evidence of drainage from acinar tissue to islets [26].

Interestingly, despite consuming a high fat diet, WT and FGF21 KO animals gained the same amount of body weight and showed the same degree of glucose tolerance; which has been previously reported [19]. Based on our previous obesity experiments, the animals were fed with high fat diet for 16 weeks. At this time point, obese and lean cohorts were sufficiently differentiated by body weight. On chow, WT and FGF21 KO had small differences in phenotype. FGF21 KO animals were slightly heavier and had impaired glucose tolerance and higher levels of circulating NEFAs, even in the fasted state (19). Interestingly, when the mice were fed HFD, this phenotypic difference in WT and FGF21 KO disappears.

A recent study suggested that pancreatic islet may be a source of systemic FGF21 as chemical ablation of pancreatic β-cells by streptozotocin resulted in a substantial reduction in circulating FGF21 [27], however this is not consistent with our finding that the majority of pancreatic FGF21 is synthesized in acinar tissue. Furthermore, another report stated that circulating FGF21 is derived from the liver because liver-specific FGF21 KO mice had undetectable circulating FGF21 [28]. Our data supports the latter study as a significant rise of FGF21 in serum is reported during fasting, at the time when expression in the pancreas is substantially reduced [1–3]. Additional studies of mouse models with tissue specific deletion of FGF21 are required to further investigate the potential contribution of each organ to the systemic circulation.

Islet cell hyperplasia has been described in FGF21 KO mice, and has been attributed to increased growth hormone signaling [29]. We assessed this possibility in our studies by measuring plasma levels of insulin like growth factor-1 (IGF-1), a key downstream target of growth hormone [30]. We found that levels were comparable between young WT and FGF21 KO animals and observed a decline in the plasma levels as the mice aged (S7 Fig), which is a known phenomenon [31]. Further, there was no significant alteration in serum IGF-1 level in mice consuming a high fat diet. Thus, at least in our model, growth hormone does not appear to play a role and mechanisms leading to the islet hyperplasia in this model remains undermined. However, it is important to note that the observed hyperplasia is similar to the other states of insulin resistance in which a circulating factor or factors are implicated but remain to be identified [32–33].

Notably, in addition to islet hyperplasia, the pancreas of obese FGF21 KO animals developed substantial periductal lymphocytic inflammation. It is known that obesity is associated with inflammation in a variety of tissues such as liver, adipose tissue, and pancreas [34–37]. Indeed, a recent study found that mice with conditional KrasG12D mutation have robust inflammation and intraepithelial lesions in the pancreas, which in this model progressed to pancreatic neoplasia [37]. Hypothesizing a similar progression in our model, we followed FGF21 KO mice for up to a year and failed to observe any additional pathology. This suggests that the immune infiltrates may contribute to pathology in circumstances under which an independent provocation serves as the initiator as in cerulean induced pancreatitis. However, it is possible that the immune infiltrates may, in part, drive the hyperplasia and hyperinsulinemia observed in the FGF21 KO mice consuming high fat diet.

Interestingly, while insulin levels are higher in FGF21 deficient mice serum glucose is significantly lower. This suggests that mechanisms beyond the requirement for control of glycemia are driving islet hyperplasia in the FGF21 KO mice consuming a high fat diet. It has been found that soluble products released by T lymphocytes can promote β- cell proliferation and islet expansion [38–39] which may contribute to islet hyperplasia in our model. Finally, we did not observe increased immune cell populations in plasma (S8 Fig), suggesting that the inflammation we observe in the pancreas is probably not systemic.

Despite the high levels of pancreatic FGF21 expression, and the notable histologic changes in both islets and periductal infiltrates in FGF21 KO mice, a key role of FGF21 in this organ seems likely but remains to be determined. In the context of reports on FGF21 in experimental pancreatitis, FGF21 has been shown to have a direct protective effect under conditions of stress [12–13]. Indeed, FGF21 has anti-inflammatory role in lipid lipotoxicity, where absence of FGF21 leads to severe hepatic steatosis on methionine choline deficient diet [5, 40] and exacerbates cardiac myopathy and oxidative stress with lipid accumulation [41–42]. Thus FGF21 may be performing an anti-inflammatory role in this tissue as well.

Although, we have shown that systemic FGF21 can signal to both the exocrine and endocrine pancreas, it is unclear how FGF21 mediates both the trophic maintenance of the islet and reduces inflammation. This may occur through direct effects of FGF21 on the pancreas or indirectly by signaling to other tissues and regulating circulating metabolites or hormones that can, in turn, regulate pancreatic function. Cell specific deletion of both FGF21 and β- klotho, the critical co-factor for FGF21 signaling, will aid in identifying the targeted effects of FGF21 *in vivo*. FGF21 is emerging as a potential human therapeutic agent for the treatment of type 2 diabetes. Therefore, implications of physiological studies across the species are required to gain a better understanding of FGF21 in the human pancreas.

## Supporting Information

S1 Fig Determining the purity of pancreatic fractions and differential expression of FGF21 receptors. (A) The purity of the islets and acinar fractions was checked by the quantitative PCR detection of Insulin and glucagon only in islets and amylase only in acinar tissue. (B) There are four different receptors for FGF21, FGFR1, FGFR2, FGFR3 and FGFR4 respectively. FGFR1 is predominant in islets while FGFR3 is predominant in acinar pancreatic fraction.
(EPS)

S2 Fig Secretion of pancreatic hormones in response to FGF21. FGF21 does not modulate insulin (A) and glucagon (B) secretion in islets isolated from wild type mice. Mouse islets were treated with 0, 10, 50 and 100nM of FGF21 at two different glucose conditions (3 and 20 mmol/l) for 60 min.
(EPS)

S3 Fig Morphometric analysis of pancreas in WT and FGF21 KO mice on chow diet. β-cell mass (A), islet cell surface area (B) in WT and FGF21 KO animals was measured. Three sections separated by >100 μm were analyzed, (n = 8 mice/group).
(EPS)

S4 Fig Analysis of fibrotic changes in pancreas. (A) Mason’s trichrome staining is represented as WT chow (i), WT HFD (ii), KO-Chow(iii), KO-HFD (iv) with no significant rise in fibrotic changes. Scale: 1 mm. (B) Pancreatic mRNA expression of the profibrotic genes were also unchanged in FGF21 KO mice fed an HFD diet compared to the WT mice such as Collagen, type I, alpha 1 (COL1A1), matrix metallopeptidase 2 (MMP-2) and transforming growth factor beta-1-like (TGFβ1).
(EPS)

S5 Fig Flow Cytometric analysis of lymphocytes. Fluorocytometric analysis of γδT cells, natural killer cells and innate lymphoid cells. Data is represented in bar graph and dot plots are not shown.
(EPS)

S6 Fig Flow cytometry analysis of myeloid cells from WT and FGF21 KO animals. Right panel: Corresponding summary data shown in bar graph. F4/80 + Macrophages (i) CD11c + dendritic cells (ii) and Ly6C + monocytes (iii) are presented. Myeloid cells are slightly higher but differences are not statistically significant. Gene expression analysis of cytokines is presented.
(EPS)

S7 Fig Plasma levels of Insulin Growth Factor -1 protein in WT and FGF21 KO animals. Chow fed animals are divided into two groups, young animals at 8–10 weeks and 24 weeks old respectively. High fat diet fed FGF21 KO animals consumed the diet for 16 weeks starting at 24 weeks of age.
(EPS)

S8 Fig Hematologic analysis of immune cells in plasma. Total and differential white cell counts from WT and FGF21 KO animals at the beginning of the experiment and at after 16 weeks of high fat diet.
(EPS)

S9 Fig Insulin tolerance test in WT and FGF21 KO animals. An insulin tolerance test in WT and FGF21 KO animals after high fat diet consumption for 16 weeks.
(EPS)
